# Cerebrospinal fluid shunt outcomes in patients with idiopathic normal pressure hydrocephalus and an Evans index below 0.3: a prospective study

**DOI:** 10.1007/s00701-026-06903-6

**Published:** 2026-05-14

**Authors:** Thanapat Akaraboonlertyot, Ekawut Chankaew, Bunpot Sitthinamsuwan, Theerapol Witthiwej, Luckchai Phonwijit, Prajak Srirabheebhat

**Affiliations:** Division of Neurosurgery, Department of Surgery, Faculty of Medicine Siriraj Hospital, Mahidol University, Bangkok, 10700 Thailand

**Keywords:** Clinical response, Evans index, Idiopathic normal pressure hydrocephalus, Lumboperitoneal shunt, Normal-sized lateral ventricle, Ventriculoperitoneal shunt

## Abstract

**Background:**

An Evans index greater than 0.3 is a key diagnostic criterion for idiopathic normal pressure hydrocephalus (iNPH). However, some patients with iNPH symptoms and an Evans index below 0.3 achieve favorable outcomes after cerebrospinal fluid (CSF) shunt surgery. This study aimed to evaluate the clinical and radiographic characteristics and CSF shunt outcomes of these patients.

**Methods:**

This prospective study enrolled patients presenting with iNPH symptoms and an Evans index below 0.3. Patients who showed clinical improvement after a spinal tap test underwent CSF shunt placement via ventriculoperitoneal or lumboperitoneal shunting. Outcomes were assessed using subjective tools and symptom-specific scores at baseline and at 3 days, 1 month, 3 months, and 6 months post-surgery.

**Results:**

Thirty-five patients with a mean (SD) Evans index of 0.26 (0.02) underwent CSF shunt placement. All clinical domains showed statistically significant improvement at 6 months compared with baseline, except cognition. Subgroup analysis revealed no statistically significant difference in preoperative and final postoperative iNPH grading scale scores between patients undergoing ventriculoperitoneal and lumboperitoneal shunting. One patient experienced shunt overdrainage causing bilateral chronic subdural hematoma that required burr hole craniostomy.

**Conclusions:**

These findings suggest that patients with iNPH-like symptoms and an Evans index below 0.3 can benefit from CSF shunt surgery. Multiple assessment tools demonstrated substantial postoperative improvement in this group. In patients with typical iNPH symptoms, a positive spinal tap response, and supportive imaging findings, an iNPH diagnosis is justified regardless of ventricular size. Such patients may be considered surgical candidates for CSF shunt placement.

**Supplementary Information:**

The online version contains supplementary material available at https://doi.org/10.1007/s00701-026-06903-6.

## Introduction

The diagnostic criteria for idiopathic normal pressure hydrocephalus (iNPH), as outlined by Ishikawa et al., classify patients into possible, probable, and definite categories [14]. Possible iNPH requires at least 1 component of the classic triad—dementia, urinary incontinence, or gait disturbance—in middle-aged to elderly patients [14]. Ventricular dilatation and narrowing of the cerebrospinal fluid (CSF) space at the high convexity on magnetic resonance imaging must also be present [14]. Although lateral ventricle size correlates with preoperative iNPH symptoms, it does not directly predict surgical outcomes following CSF shunt procedures [6].

This raises the question of why most physicians continue to rely on an Evans index greater than 0.3 for diagnosing iNPH. Some patients with hydrocephalus on brain imaging do not present with enlarged ventricles [3, 17, 37], and CSF volume on imaging does not correlate with intraventricular pressure [9, 33]. Recent studies have demonstrated that ventricular size does not correlate with clinical status, and ventricular enlargement does not reliably predict clinical improvement after CSF shunting in patients with hydrocephalus [6, 38]. Moreover, CSF shunting often does not reduce ventricular size [24].

The vast majority of patients with clinically apparent iNPH present with clearly dilated cerebral ventricles on imaging. However, this raises the question of whether dilated ventricles must be present before initiating treatment. Yamada et al. reported that frontal ventricles in iNPH patients tend to enlarge in the z-direction (vertical expansion). A strong predictor of a positive spinal tap test response is z-directional ventriculomegaly with a decreased upper-to-lower subarachnoid space ratio [38]. These findings suggest that some iNPH patients may exhibit ventriculomegaly in the z-coronal plane rather than in the x-direction (transverse axial plane), which is conventionally used to calculate the Evans index.

In clinical practice, our institute uses iNPH diagnostic guidelines that combine clinical symptoms with radiographic findings, such as an Evans index greater than 0.3 or features of disproportionately enlarged subarachnoid space hydrocephalus. These patients typically undergo CSF shunt surgery. However, we have also encountered a subset of patients who presented with symptoms of intraventricular CSF retention despite an Evans index below 0.3. Their cranial imaging revealed other features consistent with hydrocephalus, including narrowed high-convexity and medial sulci, dilated Sylvian fissures, temporal horn enlargement, periventricular edema, or a callosal angle less than 90 degrees. Some of these patients experienced symptom improvement after a spinal tap test, thereby fulfilling the probable iNPH diagnostic criteria, and several patients subsequently underwent surgery with favorable outcomes.

In the literature, Naruse et al. studied patients diagnosed with iNPH based on clinical features who had a positive spinal tap test response despite an Evans index below 0.3. After CSF shunt surgery, 9 of 14 patients (64%) experienced notable improvement of their symptoms [27].

This observation led us to prospectively collect clinical data from patients with signs of ventricular fluid retention and an Evans index below 0.3. All patients consented to post-treatment follow-up and authorized access to their information. The goal was to investigate multifaceted outcomes following CSF shunt surgery and to broaden understanding of this particular clinical scenario.

## Materials and methods

### Study design and ethical approval

This prospective study evaluated postoperative outcomes following CSF shunt surgery in patients who met the clinical criteria for iNPH. All patients showed a positive spinal tap test response but had an Evans index below 0.3 on brain imaging. Data were collected from inpatient, outpatient, and operative medical records between June 2020 and April 2022. The study was approved by the Institutional Review Board of our medical institute (Si 371/2020).

### Inclusion criteria

**Fig. 1:**
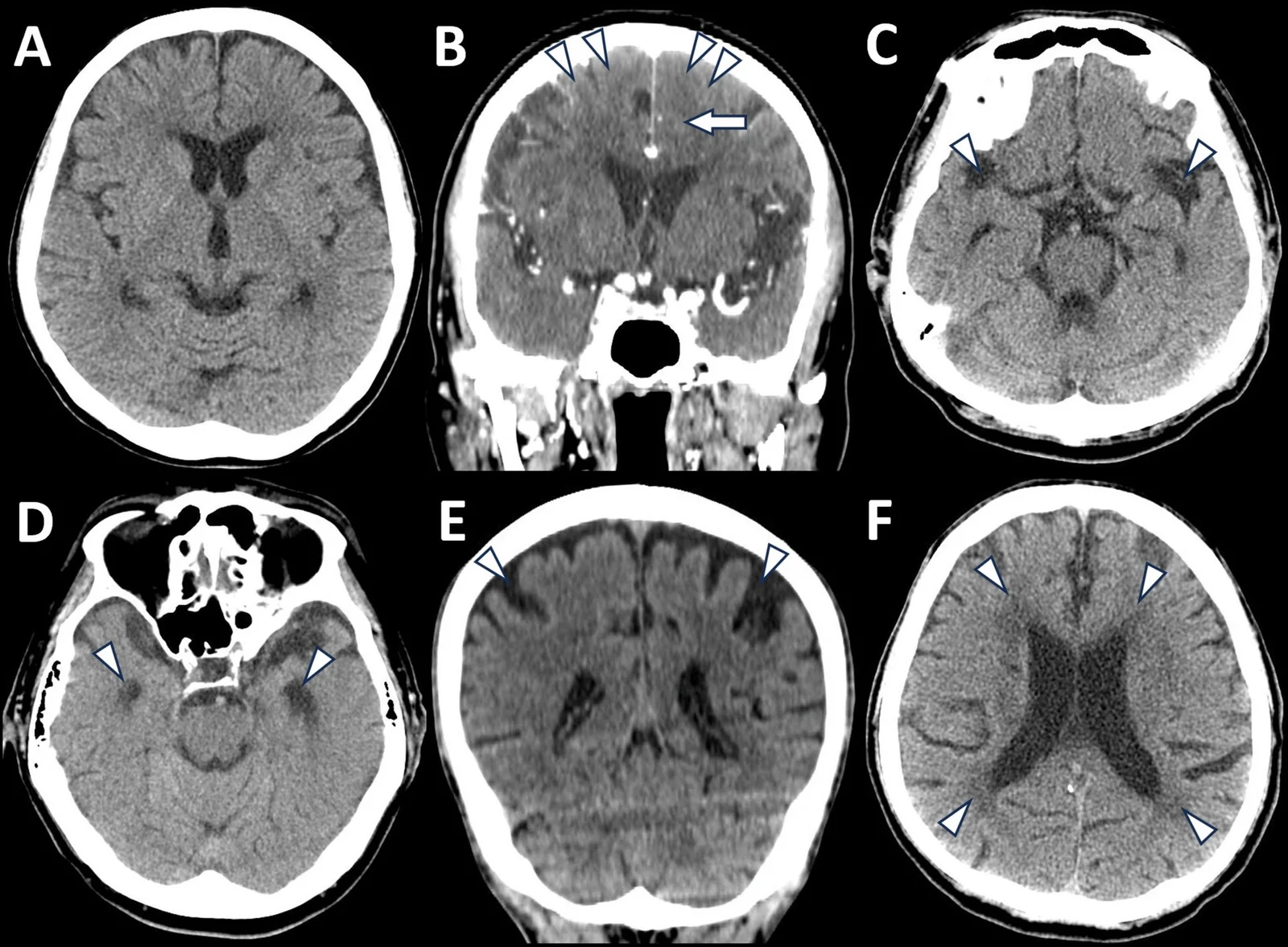
Cranial imaging features in patients presenting with symptoms of idiopathic normal pressure hydrocephalus and an Evans index below 0.3. **A** Evans index less than 0.3; **B** narrow medial sulci (arrow) and tight parasagittal cortex (double arrowheads); **C** Sylvian fissure enlargement (arrowhead); **D** temporal horn dilatation (arrowhead); **E** focally enlarged sulci (arrowhead); **F** periventricular hypodensity (arrowhead). All patients had an Evans index below 0.3 but demonstrated other radiographic hallmarks of hydrocephalus. Arrowheads and arrows indicate the relevant imaging findings in each panel
- Ability to communicate, write, and respond to the research team’s evaluations, with agreement to participate in follow-up

All patients met the following inclusion criteria:

- Age ≥ 65 years
- Presence of at least 1 cardinal symptom of iNPH, including gait disturbance, urinary incontinence, or cognitive impairment
- Clinical improvement after a spinal tap test
- Brain imaging demonstrating an Evans index less than 0.3 (Fig. 1A) with additional radiographic features compatible with hydrocephalus. These features included narrow medial sulci and tight parasagittal cortex (Fig. 1B), Sylvian fissure enlargement (Fig. 1C), temporal horn dilatation (Fig. 1D), focally enlarged sulci (Fig. 1E), and periventricular hypodensity (Fig. 1F).

### Exclusion criteria

Patients were excluded if any of the following applied:

- Incomplete data during the study period
- Concomitant diagnosis of iNPH and Parkinson disease or other movement disorders
- Secondary causes of normal pressure hydrocephalus
- Contraindications to CSF shunt surgery, such as high surgical risk, persistent thrombocytopenia or coagulopathy, or use of antiplatelet or anticoagulant medications that could not be discontinued

### CSF shunt placement procedures

Patients who met all inclusion criteria were enrolled in the study. Enrolled participants underwent CSF shunt placement via ventriculoperitoneal shunting (VPS) or lumboperitoneal shunting (LPS), depending on surgeon and patient preference.

### Follow-up schedule

All patients were evaluated at preoperative baseline and at 3 days, 1 month, 3 months, and 6 months after CSF shunt surgery.

### Shunt systems and valve management

VPS procedures were performed under general anesthesia using either a Codman Hakim programmable valve (Integra LifeSciences, Princeton, NJ, USA) or a Medtronic Strata II valve (Medtronic, Goleta, CA, USA). LPS procedures were also performed under general anesthesia using the Medtronic Strata NSC adjustable pressure valve (Medtronic). Initial valve pressure settings were determined postoperatively and adjusted as needed during follow-up visits. Shunt function and all clinical data were assessed regularly throughout follow-up.

### Outcome measurements

Outcome measurements included the following:

- *Idiopathic normal pressure hydrocephalus grading scale (iNPHGS)* [19]*:* assesses the 3 classic symptoms—cognitive impairment, gait disturbance, and urinary incontinence. Each symptom is rated from 0 to 4, with higher scores indicating more severe symptoms (Supplementary Table 1).
- *Speech (vocalization) problem assessment:* adapted from the Center for Neurologic Study–Bulbar Function Scale [30]. Scores range from 0 to 4, with higher scores reflecting more severe vocalization impairment (dysphonia; Supplementary Table 2).
- *Swallowing problem assessment:* adapted from the Center for Neurologic Study–Bulbar Function Scale [31]. Scores range from 0 to 4, with higher scores indicating more severe swallowing difficulty (Supplementary Table 3).
- *Balance on standing problem assessment:* adapted from the Hellström iNPH Scale [13]. Scores range from 1 to 7, with higher scores indicating more severe balance impairment (Supplementary Table 4).
- *Emotion problem assessment:* adapted from the Bipolar UK Mood Scale [32]. Scores range from 0 to 4, with higher scores indicating more severe emotional problems (Supplementary Table 5).
- *Dizziness problem assessment:* adapted from the AMER Dizziness Diagnostic Scale [2]. Scores range from 0 to 4, with higher scores indicating more severe dizziness (Supplementary Table 6).
- *Barthel Index:* used to assess ability in performing basic activities of daily living [20], adapted by Collin and Wade [7, 36]. Scores range from 0 to 20 points, with higher scores indicating greater functional ability (Supplementary Table 7).
- *Thai Mental State Examination (TMSE) for cognitive function assessment* [34]*:* evaluates 6 cognitive domains—orientation, registration, attention, calculation, language, and recall. Total scores range from 0 to 30, with scores ≤ 23 indicating possible cognitive impairment. A cutoff score of 23 has demonstrated a sensitivity of 68.5%–82% and a specificity of 63%–88% for detecting mild cognitive impairment [26]. This scoring system is widely used to evaluate cognitive function in Thai patients; however, no English version is currently available.
- *Three-meter Timed Up and Go (TUG) test* [28] *for gait assessment:* measures the total number of walking steps, number of turning steps, and total time taken.

### Assessment schedule

The iNPHGS, speech (vocalization), swallowing, balance on standing, emotion, and dizziness assessments were evaluated at preoperative baseline and at 3 days, 1 month, 3 months, and 6 months postoperatively. The Barthel Index was assessed at all time points except 3 months. The TMSE and video-recorded 3-m TUG test were conducted at preoperative baseline, 3 days, and 6 months postoperatively.

### Definitions of improvement

Gait improvement was defined as a reduction in the number of steps or total time taken to complete the 3-m TUG test. Improvement of other symptoms was defined as an improvement of at least 1 point in any of the following: TMSE, iNPHGS, and the assessments for balance, vocalization, swallowing, emotion, dizziness, and the Barthel Index.

### Statistical analysis

Descriptive statistics were used to summarize demographic and clinical characteristics of the study population. Quantitative data were presented as mean (SD) or median (IQR), as appropriate, and qualitative data were expressed as number and percentage.

### Comparisons across time points

The Friedman test was used to evaluate differences in ordinal scale outcomes across multiple time points: pre-surgery and at 3 days, 1 month, 3 months, and 6 months postoperatively. The McNemar test was used to assess differences in nominal variables between baseline and 6-month follow-up. Paired *t* tests and repeated-measures analysis of variance were used to determine differences in numerical variables over time.

### Pairwise and subgroup comparisons

Pairwise comparisons were conducted using the Bonferroni correction to identify statistically significant differences between pre-surgery and each postoperative time point (3 days, 1 month, 3 months, and 6 months). For subgroup analysis, iNPHGS scores were compared between patients undergoing VPS and LPS using independent-sample *t* tests and analysis of covariance for ordinal scales.

### Comparison of variables between patients with and without clinical improvement following CSF shunt surgery

Based on the iNPHGS at 6 months after CSF shunt surgery, all patients were classified into the groups with and without clinical improvement. A comparative analysis of variables between both outcome groups was performed to identify factors associated with CSF shunt unresponsiveness. The categorical data were analyzed using the chi-square test or Fisher’s exact test. The numeric data were compared between both groups of patients using the independent t-test or Mann–Whitney U test.

### Statistical software and significance threshold

A 95% CI was used to indicate the strength and direction of associations. All data were recorded and analyzed using PASW Statistics version 18 (SPSS Inc, Chicago, IL, USA). All tests were 2-sided, and a *P* value less than 0.05 was considered statistically significant.

## Results

### Study population

This prospective cohort study included all patients presenting with symptoms consistent with iNPH who had an Evans index below 0.3 but showed other radiological signs of hydrocephalus. Most patients exhibited at least 1 symptom of the classic iNPH triad, and some also experienced bulbar symptoms (vocalization or swallowing dysfunction), emotional instability, or dizziness.

### Demographic and clinical characteristics

**Fig. 2:**
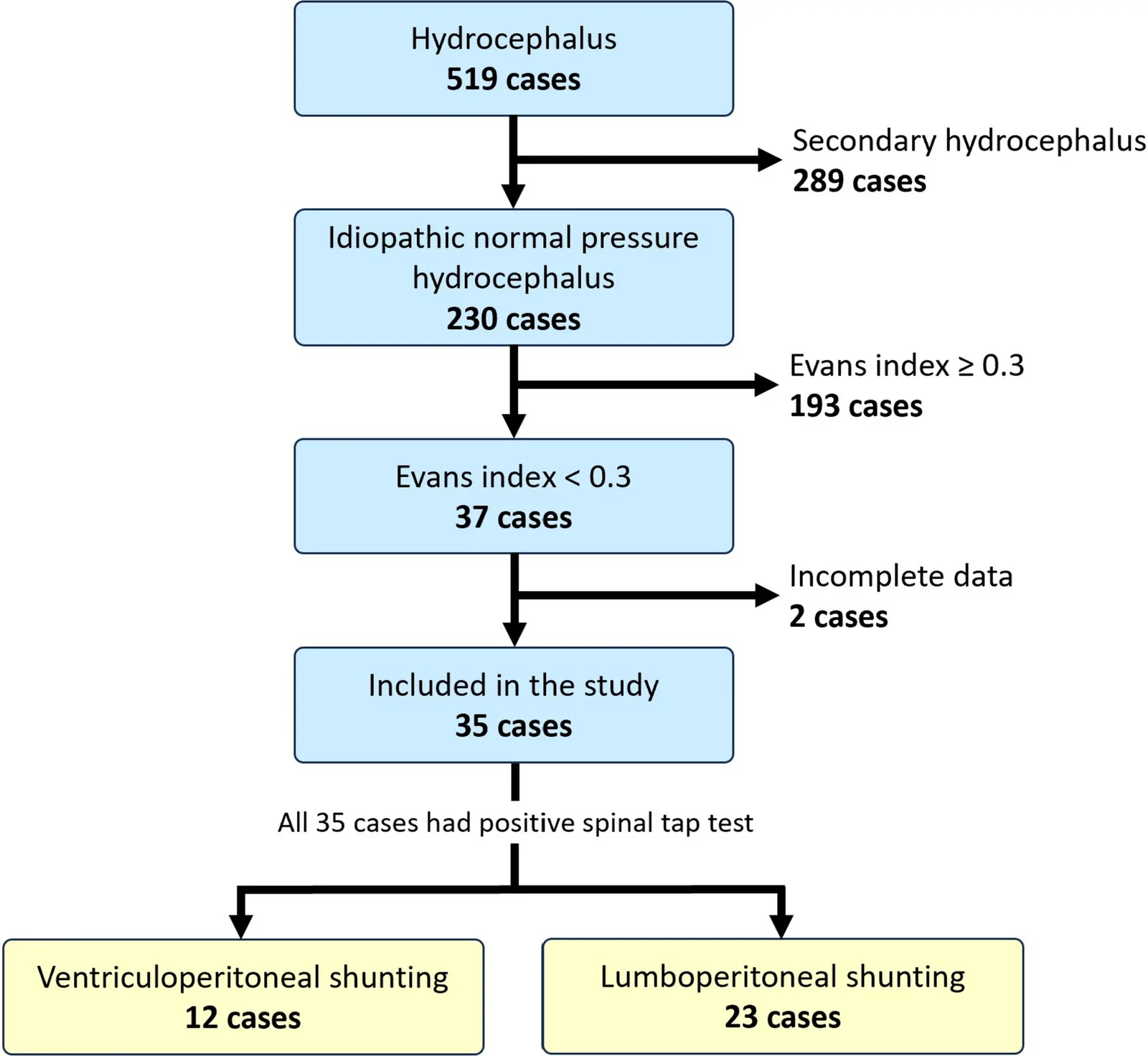
The algorithm of the study process

Of 37 patients who met the inclusion criteria, 2 with incomplete follow-up data were excluded, leaving 35 patients for analysis. The algorithm of the study process is shown in Fig. 2. Mean (SD) age was 75.8 (7.2) years; 23 patients (65.7%) were male and 12 (34.3%) were female.

### Preoperative symptom profile

Regarding the classic triad of iNPH, gait disturbance was present in all 35 patients (100%), cognitive impairment in 34 (97.1%), and urinary incontinence in 32 (91.4%). Thirty-one patients (88.6%) presented with all 3 components of the classic triad. Bulbar dysfunction (vocalization or swallowing impairment) was a common associated symptom, found in 31 patients (88.6%). Notably, 34 patients (97.1%) had emotional problems on preoperative assessment. Twenty patients (57.1%) had cognitive impairment detected by TMSE, with a total score of 23 or less On the 3-m TUG test, 34 patients (97.1%) walked more slowly than the standard cutoff. Patient demographic and clinical characteristics are summarized in Table 1.

**Table 1:** Demographic and clinical characteristics of the study population (*N* = 35)

Variable	Result
Age, y, mean (SD)	75.8 (7.2)
Sex, *n* (%)	
Male	23 (65.7)
Female	12 (34.3)
Core symptoms, *n* (%)	
Classic triad	
Gait disturbance	35 (100)
Cognitive impairment	34 (97.1)
Urinary incontinence	32 (91.4)
Bulbar dysfunction	31 (88.6)
Complete classic triad	31 (88.6)
Complete classic triad plus bulbar dysfunction	30 (85.7)
Dizziness	14 (40)
Emotional dysfunction	34 (97.1)
Objective evaluation, *n* (%)	
TMSE score ≤ 23 points	20 (57.1)
3-m TUG test slower than 13.5 s	34 (97.1)
CSF shunt type, *n* (%)	
Programmable VPS	12 (34.3)
Programmable LPS	23 (65.7)
Complications during follow-up, *n* (%)	
Shunt-related complication	1 (2.9)
Non–shunt-related complication	3 (8.6)
Mortality (non–shunt-related)	1 (2.9)

Values are presented as mean (SD) or *n* (%)

*CSF* Cerebrospinal fluid, *LPS* Lumboperitoneal shunting, *n* number of patients, *SD* Standard deviation, *TMSE* Thai Mental State Examination, *TUG* Timed Up and Go, *VPS* Ventriculoperitoneal shunting

### Radiographic characteristics

Radiographic characteristics are presented in Table 2. All patients had an Evans index below 0.3, with a mean (SD) of 0.26 (0.02). Common hallmarks of hydrocephalus—narrow sulci at the parafalcine and vertex regions, temporal horn enlargement, and periventricular hypodensity indicating interstitial edema—were present in all patients. Most patients showed enlarged Sylvian fissures (88.6%) and focally enlarged sulci (94.3%), whereas a narrow callosal angle was rare (8.6%). Mean (SD) callosal angle was 129.1 (8.4) and 106.1 (11.3) degrees at the anterior and posterior parts of the corpus callosum, respectively.

**Table 2:** Radiographic characteristics of the study population (*N* = 35)

Variable	Result
Evans index	
Evans index < 0.3, *n* (%)	35 (100)
Evans index, mean (SD)	0.26 (0.02)
Narrow sulci at parafalcine and vertex regions, *n* (%)	35 (100)
Enlarged Sylvian fissure, *n* (%)	31 (88.6)
Focally enlarged sulci, *n* (%)	33 (94.3)
Temporal horn	
Enlargement, *n* (%)	35 (100)
Width, mm, mean (SD)	7.2 (1.6)
Callosal angle	
Narrow callosal angle (< 90°), *n* (%)	3 (8.6)
Anterior callosal angle^a^, degrees, mean (SD)	129.1 (8.4)
Posterior callosal angle^b^, degrees, mean (SD)	106.1 (11.3)
Periventricular hypodensity, *n* (%)	35 (100)
Radscale score^c^, mean (SD)	7.7 (1.2)

Values are presented as mean (SD) or *n* (%)

^a^ Measured on a coronal slice at a right angle to the AC–PC line passing through the AC

^b^ Measured on a coronal slice at a right angle to the AC–PC line passing through the PC

^c^ Radscale is a radiological scale used to quantify the degree of hydrocephalus based on imaging features; higher scores indicate more pronounced hydrocephalic changes

*AC* Anterior commissure, *AC–PC* Anterior commissure–posterior commissure, *n* number of patients, *PC* Posterior commissure, *SD* Standard deviation

### CSF shunt procedures

All patients showed clinical improvement after a spinal tap test and subsequently underwent CSF shunt surgery. Twelve patients (34.3%) underwent VPS, and LPS was performed in the remaining 23 (65.7%).

### Postoperative clinical outcomes

Clinical improvement following surgery was assessed using individual measurement tools, with results detailed in Table 3. At 6 months postoperatively, statistically significant improvement was observed in gait score on the iNPHGS (*P* < 0.001), urination score on the iNPHGS (*P* = 0.001), vocalization (*P* < 0.001), swallowing (*P* < 0.001), balance (*P* < 0.001), emotion (*P* < 0.001), dizziness (*P* < 0.001), activities of daily living assessed by the Barthel Index (*P* = 0.030), cognitive function assessed by TMSE (*P* < 0.001), walking steps (*P* < 0.001), turning steps (*P* < 0.001), and walking speed (*P* < 0.001) assessed by the 3-m TUG test. However, the cognition score on the iNPHGS showed no statistically significant improvement (*P* = 0.969) at 6 months after CSF shunt surgery.

**Table 3:** Comparison of preoperative and postoperative clinical scores across assessment tools

Score measurement	*n*	Score					*P* value	Improved^d^, *n* (%)
		Preop	3 d	1 mo	3 mo	6 mo		
iNPHGS, median (IQR)	35	7 (5–8)	5 (4–7)	4 (2–7)	4 (2–7)	4 (2–6)	< 0.001^e^	29 (82.9)
Gait, median (IQR)	35	2 (2–3)	1 (1–2)	1 (0–2)	1 (0–2)	1 (0–2)	< 0.001^e^	27 (77.1)
Cognition, median (IQR)	34	2.5 (2–3)	2 (2–3)	2 (2–3)	1.75 (2–3)	1 (1–3)	0.969	9 (26.5)
Urination, median (IQR)	32	2 (1–3)	1.5 (1–2.8)	1 (0.5–2)	1 (0–2)	1 (0–2)	< 0.001^e^	17 (53.1)
Speech (bulbar)^a^, median (IQR)	31	2 (2–2)	1 (1–2)	1 (0–1)	1 (0–1)	1 (0–1)	< 0.001^e^	29 (93.5)
Swallowing (bulbar)^b^, median (IQR)	31	2 (1–2)	2 (1–2)	1 (0–1)	0 (0–1)	0 (0–1)	< 0.001^e^	28 (90.3)
Balance on standing^c^, median (IQR)	35	5 (4–6)	4 (4–5)	3 (2–4.5)	3 (2–4)	3 (2–4)	< 0.001^e^	29 (82.9)
Emotion^f^, median (IQR)	34	2 (2–2)	2 (1–2)	1 (1–2)	1 (1–2)	1 (1–2)	< 0.001^e^	23 (67.6)
Dizziness^g^, median (IQR)	14	2 (2–3)	1 (0–1)	1 (0–1)	1 (0–1)	0.5 (0–1)	< 0.001^e^	14 (100)
Barthel Index^h^, mean (SD)	35	14.2 (0.9)	16.3 (1.1)	16.5 (1.1)	–	16 (1.1)	0.030^e^	29 (82.6)
TMSE^i^, mean (SD)	35	21.8 (1.4)	24.1 (1.3)	–	–	23.2 (1.4)	< 0.001^e^	10/19 (52.6)^j^
3-m TUG test								27 (77.1)
Walking steps, mean (SD)	35	31.4 (14.2)	30.7 (13.7)	–	–	22.9 (11.5)	< 0.001^e^	–
Turning steps, mean (SD)	35	6 (4.3)	5.2 (3.2)	–	–	5.2 (3.3)	< 0.001^e^	–
Time (seconds), mean (SD)	35	37.1 (26.7)	33.7 (24.6)	–	–	23 (14.3)	< 0.001^e^	–

*P* values for preoperative vs 6-month postoperative comparisons. En dash (–) indicates assessment not performed at that time point

^a^ Assessed by speech (vocalization) problem assessment (scored 0–4)

^b^ Assessed by swallowing problem assessment (scored 0–4)

^c^ Assessed by balance on standing problem assessment (scored 1–7)

^d^ Number of patients with improvement at 6 months postoperatively

^e^ Statistically significant improvement (*P* < 0.05)

^f^ Assessed by emotion problem assessment (scored 0–4)

^g^ Assessed by dizziness problem assessment (scored 0–4)

^h^ Assessed by the Barthel Index (scored 0–20); higher scores indicate greater functional independence

^i^ Assessed by the Thai Mental State Examination (scored 0–30); scores ≤ 23 indicate possible cognitive impairment

^j^ Of 19 patients with preoperative TMSE scores ≤ 23, 10 (52.6%) achieved scores > 23 at 6 months postoperatively

*d* day(s), *iNPHGS* idiopathic normal pressure hydrocephalus grading scale, *IQR* Interquartile range, *mo* month(s), *n* number of patients, *P* probability value; postop, postoperative; preop, preoperative, *SD* standard deviation, *TMSE* Thai Mental State Examination, *TUG* Timed Up and Go

### Subgroup comparison of shunt types

Both VPS and LPS demonstrated equivalent effectiveness as assessed by the iNPHGS (Table 4).

**Table 4:** Comparison of iNPHGS scores between patients undergoing ventriculoperitoneal and lumboperitoneal shunting

Time point	VPS (*n* = 12)	LPS (*n* = 23)	Difference (95% CI)	*P* value
Preoperative, mean (SD)	7.3 (1.8)	6.6 (2.1)	0.8 (–0.7 to 2.2)	0.286
Postoperative at 6 mo, mean (SD)	5.3 (1.5)	4.4 (1.3)	0.8 (–0.8 to 2.5)	0.295

iNPHGS scores are presented as mean (SD). No statistically significant differences were observed between shunt types at either time point

*CI* Confidence interval, *iNPHGS* Idiopathic normal pressure hydrocephalus grading scale, *LPS* Lumboperitoneal shunting, *mo* month(s), *n* number of patients, *P* probability value, *SD* Standard deviation, *VPS* Ventriculoperitoneal shunting

### Overall clinical improvement

At 6 months postoperatively, 29 patients (82.9%) showed clinical improvement as measured by the iNPHGS. The most notable improvement was observed in gait function, with some patients demonstrating marked improvement in gait and bulbar (vocalization) symptoms as early as postoperative day 3. Clinical improvements in gait were supported by both iNPHGS scores and results from the 3-m TUG test.

### Timeline of symptom improvement

**Fig. 3:**
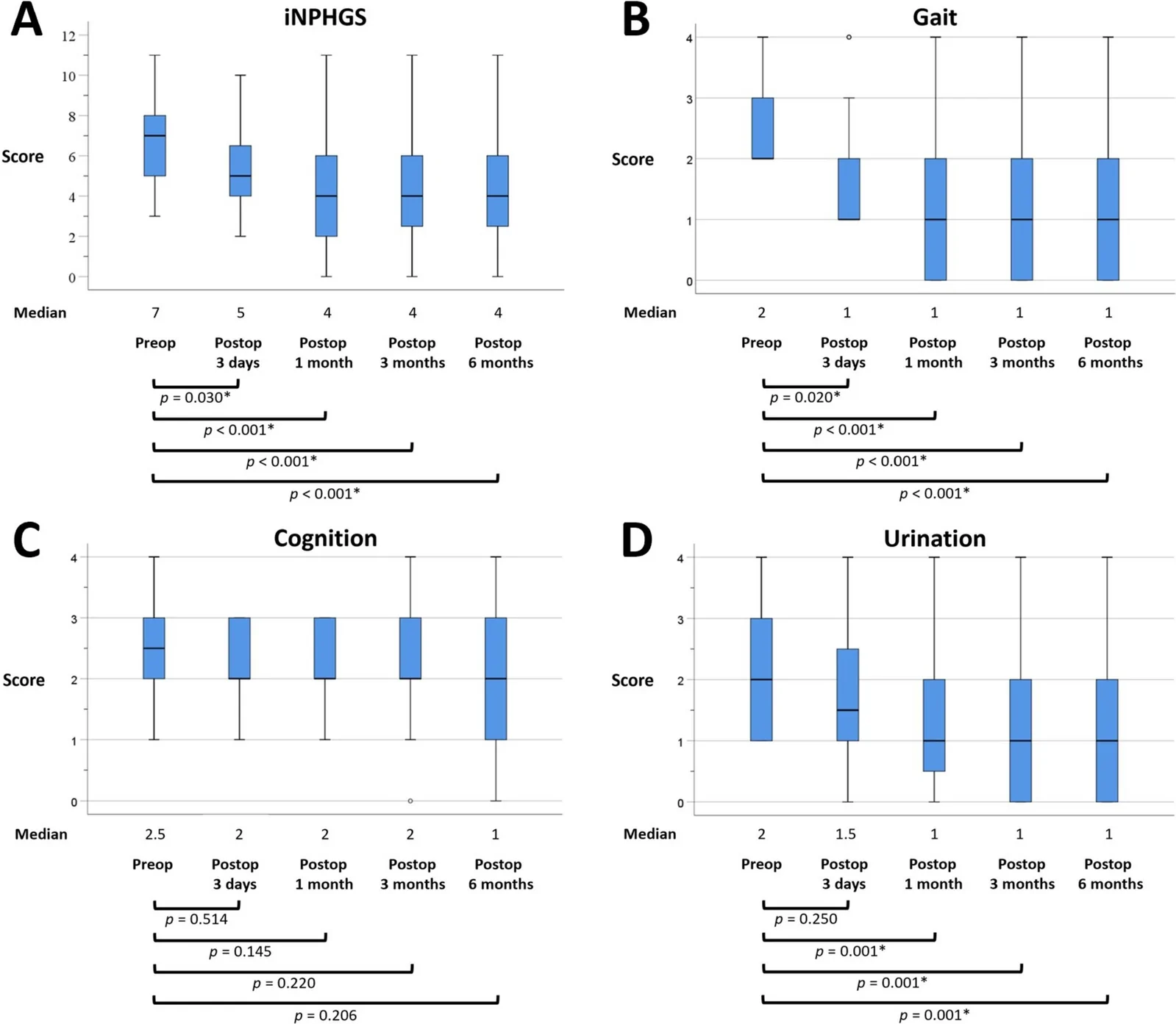
Idiopathic normal pressure hydrocephalus grading scale (iNPHGS) scores at preoperative baseline and postoperative time points following CSF shunt surgery. **A** Total iNPHGS score; **B** gait domain; **C** cognition domain; **D** urination domain. Asterisks indicate statistically significant improvement compared with baseline (*P* < 0.05). The total iNPHGS score and scores for all domains except cognition showed statistically significant improvement after surgery. Lower scores indicate less severe symptoms. Error bars represent variability around the central tendency. Abbreviations: CSF, cerebrospinal fluid; iNPHGS, idiopathic normal pressure hydrocephalus grading scale; *P*, probability value; postop, postoperative; preop, preoperative

**Fig. 4:**
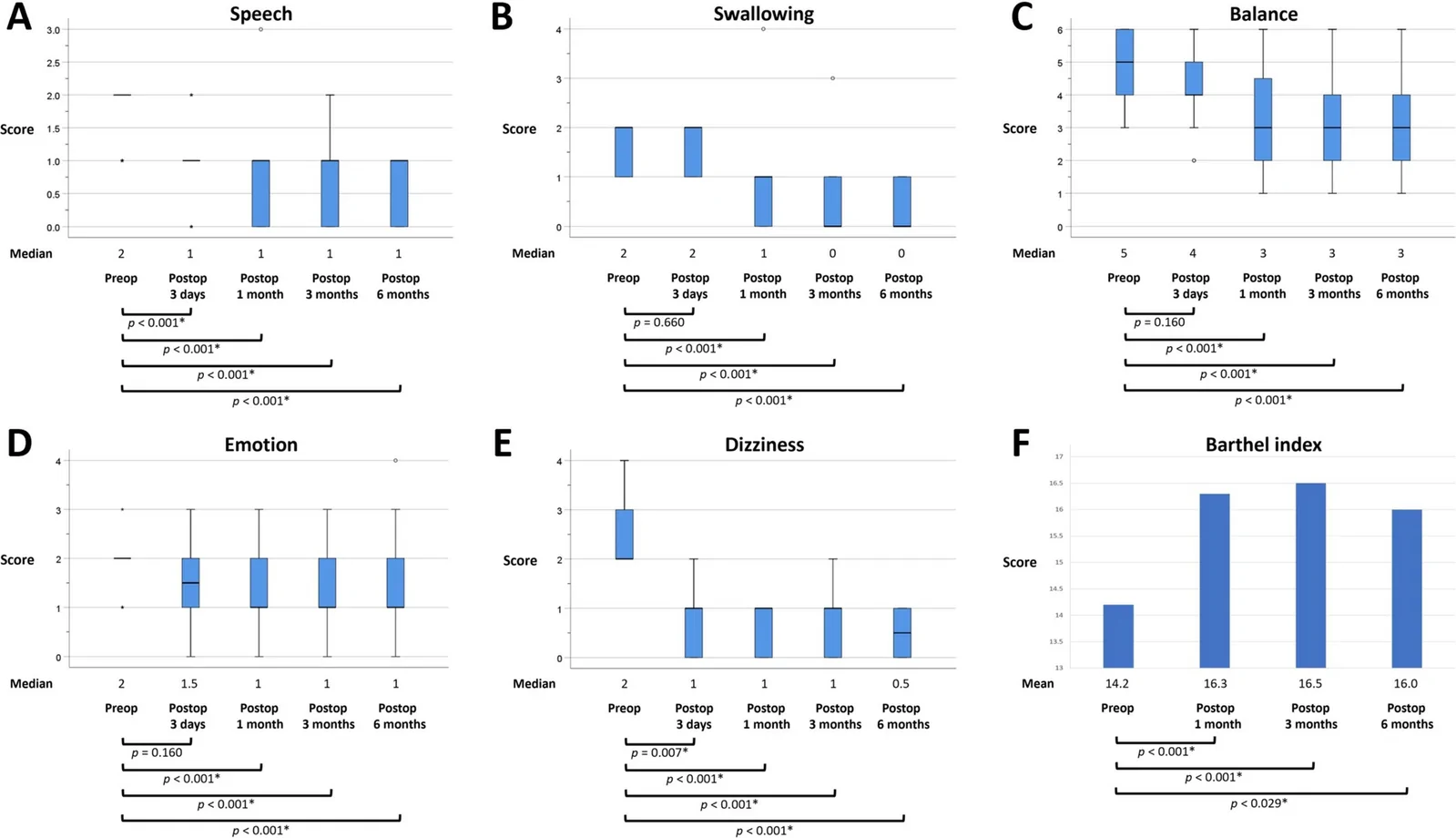
Clinical outcome scores at preoperative baseline and postoperative time points following CSF shunt surgery. **A** Speech (vocalization); **B** swallowing; **C** balance on standing; **D** emotion; **E** dizziness; **F** Barthel Index. Asterisks indicate statistically significant improvement compared with baseline (*P* < 0.05). Statistically significant postoperative improvement was observed in all parameters shown. For panels (**A**)–(**E**), lower scores indicate less severe symptoms. For panel (**F**), higher scores indicate greater functional independence. Error bars represent variability around the central tendency. Abbreviations: CSF, cerebrospinal fluid; *P*, probability value; postop, postoperative; preop, preoperative

**Fig. 5:**
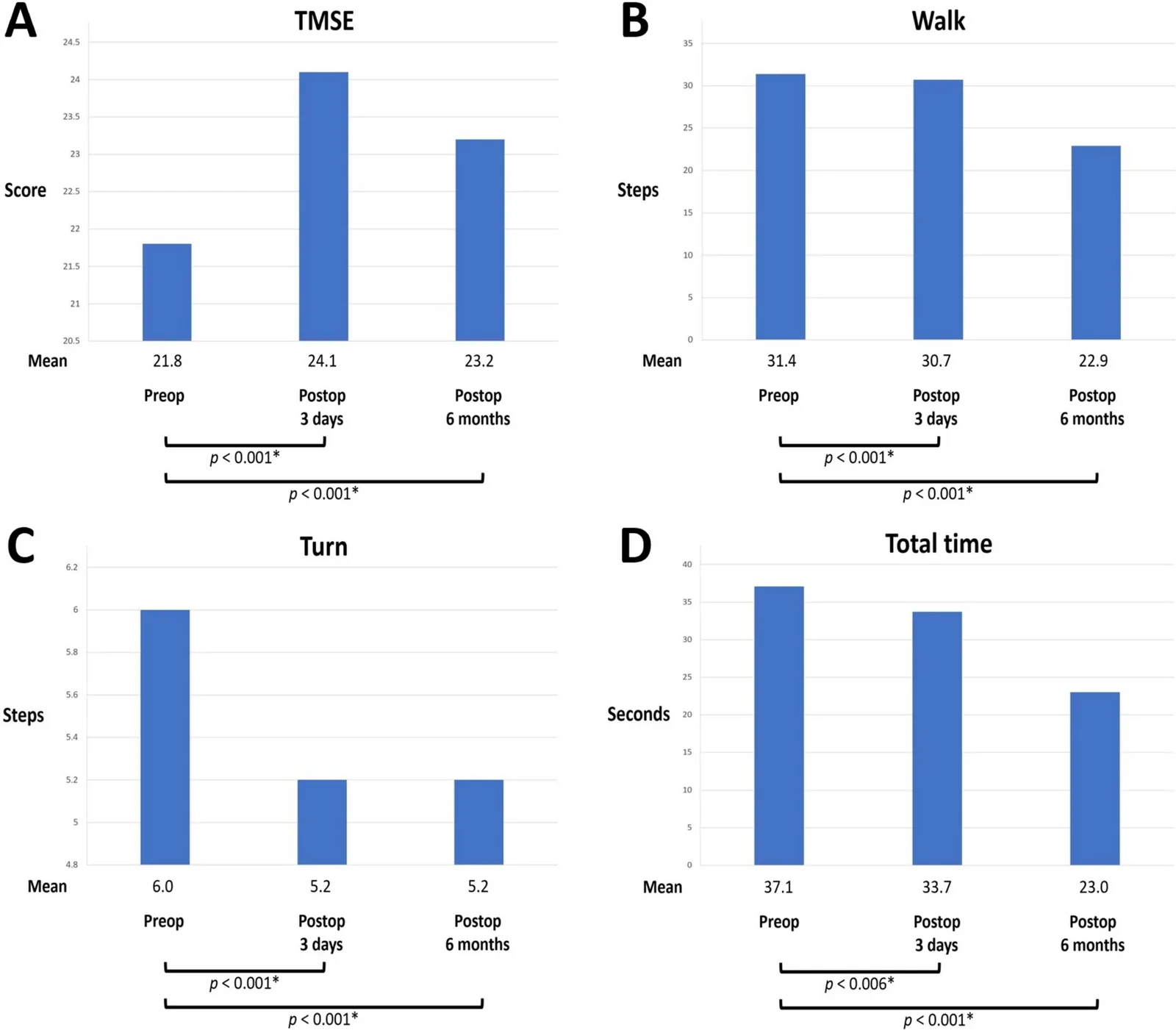
Thai Mental State Examination (TMSE) scores and 3-m Timed Up and Go (TUG) test results at preoperative baseline and postoperative time points following CSF shunt surgery. **A** TMSE score; **B** walking steps; **C** turning steps; **D** total time. Asterisks indicate statistically significant improvement compared with baseline (*P* < 0.05). Statistically significant postoperative improvement was observed in TMSE scores and all TUG test domains. For panel (**A**), higher TMSE scores indicate better cognitive function (scores ≤ 23 suggest possible cognitive impairment). For panels (**B**)–(**D**), lower values indicate improved gait performance. Error bars represent variability around the central tendency. Abbreviations: CSF, cerebrospinal fluid; *P*, probability value; postop, postoperative; preop, preoperative; TMSE, Thai Mental State Examination; TUG, Timed Up and Go

Gait improvement persisted throughout the follow-up period, with the greatest therapeutic benefit observed by 1 month after surgery. Delayed improvements in urination, balance, bulbar (swallowing), and emotional symptoms emerged at approximately 1 month after CSF shunt surgery and remained stable through 6 months. At 6-month follow-up, 90% of patients with preoperative swallowing dysfunction achieved full recovery, allowing nasogastric tube removal and resumption of a regular diet. Cognition showed the least improvement according to iNPHGS scores; however, TMSE scores demonstrated statistically significant improvement. Details of clinical outcomes following CSF shunt surgery are presented in Figs. 3, 4, 5.

### Factors associated with CSF shunt unresponsiveness

Regarding the results of iNPHGS at 6 months after the shunt surgery, 29 (82.9%) patients had clinical improvement, while the remaining 6 (17.1%) showed no clinical improvement. In a comparison of variables between the groups with and without clinical improvement (Table 5), the total iNPHGS score of the group with clinical improvement was significantly lower than that of the counterpart (median total score 7 vs. 9, respectively; p = 0.040). Gait disturbance score was an isolated symptom parameter showing a statistically significant difference between the groups, whereas cognitive impairment and urinary incontinence scores were not.

**Table 5:** Comparison of variables between patients with and without clinical improvement assessed by iNPHGS at 6 months following CSF shunt surgery

Variable	Patients with clinical improvement (*n* = 29)	Patients without clinical improvement (*n* = 6)	*p*-value
Outcome at 6 months following CSF shunt surgery			
iNPHGS score, median (IQR)			
Total score	7 (5–7.5)	9 (6–10)	0.040^a^
Gait disturbance score	2 (2–3)	3.5 (2–4)	0.006^a^
Cognitive impairment score	2 (2–3)	3 (3–3)	0.125
Urinary incontinence score	2 (1–3)	2.5 (1–4)	0.199
Demographic and clinical characteristics			
Age, years, mean (SD)	75.6 (7)	77.2 (7.4)	0.624
Sex, *n* (%)			
Male	19 (65.5)	4 (66.7)	1.000
Female	10 (34.5)	2 (33.3)	
Symptoms, *n* (%)			
Classic triad			
Gait disturbance	29 (100)	6 (100)	1.000
Cognitive impairment	28 (96.6)	6 (100)	1.000
Urinary incontinence	26 (92.9)	6 (100)	1.000
Bulbar dysfunction	26 (92.9)	5 (83.3)	1.000
Complete classic triad	25 (86.2)	6 (100)	0.581
Complete classic triad plus bulbar dysfunction	25 (86.2)	5 (83.3)	1.000
Dizziness	13 (44.8)	1 (16.7)	0.366
Emotional dysfunction	28 (96.6)	6 (100)	1.000
Objective evaluation			
TMSE score, median (IQR)	23 (21–28)	17.5 (13–24)	0.084
TMSE score ≤ 23 points, n (%)	16 (55.2)	4 (66.7)	0.680
3-m TUG test slower than 13.5 s, n (%)	29 (100)	5 (83.3)	0.171
Radiographic characteristics			
Evans index, mean (SD)	0.258 (0.017)	0.262 (0.009)	0.641
Narrow sulci at parafalcine and vertex regions, *n* (%)	29 (100)	6 (100)	1.000
Enlarged Sylvian fissure, *n* (%)	25 (86.2)	6 (100)	1.000
Focally enlarged sulci, *n* (%)	27 (93.1)	6 (100)	1.000
Temporal horn			
Enlargement, *n* (%)	29 (100)	6 (100)	1.000
Width, mm, mean (SD)	7.1 (1.7)	7.8 (1.4)	0.369
Callosal angle			
Narrow callosal angle (< 90°), *n* (%)	2 (6.9)	1 (16.7)	0.999
Anterior callosal angle^b^, degrees, mean (SD)	129.3 (9)	128.5 (5.3)	0.844
Posterior callosal angle^c^, degrees, mean (SD)	107.8 (11.3)	98 (7.1)	0.055
Periventricular hypodensity, *n* (%)	29 (100)	6 (100)	1.000
Radscale score^d^, mean (SD)	7.6 (1.2)	8.2 (0.9)	0.318
Treatment characteristics			
CSF shunt type, *n* (%)			
Programmable VPS	10 (34.5)	2 (33.3)	1.000
Programmable LPS	19 (65.5)	4 (66.7)	
Complications during follow-up, *n* (%)			
Shunt-related complication	1 (3.4)	0 (0)	1.000
Non–shunt-related complication	2 (6.9)	1 (16.7)	0.999
Mortality (non–shunt-related)	1 (3.4)	0 (0)	1.000

Values are presented as mean (SD), median (IQR) or *n* (%)

^a^ Indicates statistically significant difference between both groups

^b^ Measured on a coronal slice at a right angle to the AC–PC line passing through the AC

^c^ Measured on a coronal slice at a right angle to the AC–PC line passing through the PC

^d^ Radscale is a radiological scale used to quantify the degree of hydrocephalus based on imaging features; higher scores indicate more pronounced hydrocephalic changes

*AC* anterior commissure, *AC–PC* anterior commissure–posterior commissure, *CSF* Cerebrospinal fluid, *iNPHGS* Idiopathic normal pressure hydrocephalus grading scale, *IQR* Interquartile range, *LPS* Lumboperitoneal shunting, *n* number of patients, *PC* Posterior commissure, *SD* Standard deviation, *TMSE* Thai Mental State Examination, *TUG* Timed Up and Go, *VPS* Ventriculoperitoneal shunting

Other variables, including demographic, clinical, radiographic, and treatment factors, were not significantly different between the groups. These results could be summarized as no variable was found to be a factor associated with CSF shunt unresponsiveness in NPH patients who had an Evans index below 0.3.

### Complications and mortality

Four patients experienced complications during the follow-up period: 3 developed medical complications (ischemic stroke, sepsis), and 1 developed a surgical complication. The surgical complication was bilateral subdural hematoma caused by CSF shunt overdrainage following VPS placement at 6 months, which required bilateral burr hole craniostomies and hematoma drainage. Additionally, 1 patient died 6 months postoperatively from a cause unrelated to shunt surgery.

## Discussion

Clinical symptoms and signs of hydrocephalus in individuals with an Evans index below 0.3 may represent an early-stage manifestation of iNPH, characterized by elevated intraventricular pressure without proportional ventricular dilatation. Our findings support those of a previous study [27], which reported postoperative improvement in 14 patients diagnosed with iNPH despite an Evans index of 0.3 or less, 9 of whom showed clinical improvement. The pathophysiological basis of this phenomenon lies in age-related reductions in brain compliance (increased parenchymal stiffness), which limits ventricular expansion despite pressure changes. In elderly patients, the brain exhibits heightened pressure–volume sensitivity, and even minimal increases in intracranial volume can result in considerable elevations in intracranial pressure. Traditional reliance on ventricular size metrics may therefore be insufficient for surgical decision-making. Delaying intervention until ventricular enlargement is visible on imaging risks irreversible neurological damage, and pressure normalization should ideally precede overt structural changes.

In earlier studies of patients with enlarged ventricles, the classic iNPH triad appeared in the following frequencies: gait disturbance in 94%–100% of cases, cognitive impairment in 78%–98%, and urinary dysfunction in 76%–83% [10, 12, 18, 22, 25]. All 3 symptoms were present simultaneously in approximately 60% of cases. In our study, the incidence of gait disturbance, cognitive impairment, and urinary dysfunction was 100%, 97.1%, and 91.4%, respectively, with the complete triad present in 88.6% of patients.

Our surgical outcomes were also comparable with previous findings. Past studies reported improvement rates following VPS of 80%–88% for gait, 30%–65% for cognition, and 30%–56% for urinary incontinence [4, 11, 23, 29]. In our cohort, improvement rates were 77.1% for gait, 26.5% for cognitive function, and 53.1% for urinary incontinence.

In our study, gait disturbance, vocal dysfunction, and dizziness showed early improvement as soon as 3 days postoperatively, whereas balance, urination, swallowing, and emotional symptoms showed delayed improvement, typically by 1 month after surgery. These findings are consistent with a systematic review by Marmarou et al. [21], which found that gait improvements may occur within 1 or 2 days after a CSF tap test, while improvements in cognition and urination may take up to 1 week—a finding supported by other studies [15, 19]. Notably, our study observed improvement in balance in 29 of 35 patients (82.9%) concurrently with gait improvement (27 of 35; 77.1%), suggesting that balance can improve simultaneously with gait in this patient population.

In a study by Kubo et al. [19], patient symptoms were assessed using the iNPHGS. Pre-shunt iNPHGS scores were similar between their cohort and ours; however, the postoperative improvement in iNPHGS scores in our study was notably greater. This difference may be attributed to the fact that our patients were diagnosed at an earlier stage of iNPH, with smaller ventricular sizes compared with patients with fully developed iNPH. This finding supports the concept that early diagnosis and treatment can result in greater clinical responsiveness.

Regarding cognitive assessment, the TMSE proved to be more sensitive than the iNPHGS in detecting cognitive changes; we therefore recommend using both tools for a more comprehensive evaluation. In our study, 1 patient who had previously been diagnosed with Alzheimer disease showed statistically significant cognitive improvement following CSF shunt surgery. This supports the concept that iNPH can present as a potentially reversible cause of dementia and should therefore be ruled out in all dementia cases.

A previous case report described a patient presenting with chronic dizziness, postural instability, and gait dysfunction [35]. Imaging revealed hydrocephalus, and the patient underwent endoscopic third ventriculostomy, which led to subjective improvement in dizziness. An additional case of a young male patient with atypical manifestations of normal pressure hydrocephalus was reported [1]. He presented with dizziness and mild ataxia, and cranial imaging revealed global ventricular enlargement. The prevalence of dizziness in iNPH patients has not been well characterized in the literature; in the present study, it was 40%, and all affected patients (100%) showed symptom improvement following CSF shunt surgery. Typically, dizziness in our patients resolved within a few days after surgery. These results suggest that vestibular dysfunction could be an underrecognized component of iNPH.

We propose a potential mechanism to explain this phenomenon. Fluctuating CSF pressure may transmit through the cochlear aqueduct into the endolymphatic system, generating a pressure gradient between the endolymph and perilymph. This differential pressure could displace the Reissner membrane, resulting in vestibular impairment.

Our study also identified bulbar symptoms (swallowing and vocal dysfunction) as notable clinical manifestations of iNPH, consistent with the findings of Chankaew et al. [5]. In our cohort, swallowing impairment was present in 89% of patients compared with 86% in the previous study. Speech and vocal impairment were found in 89% of patients, compared with 67%–75.5% in prior studies. In an earlier study, bulbar function was measured using a nasometer, which requires otolaryngologists and specialized equipment, limiting its practicality for routine clinical use [39]. To address this limitation, our study proposes an adapted version of the Center for Neurologic Study–Bulbar Function Scale as a reliable, practical assessment tool. This scale was specifically designed to assess both swallowing and vocal function in iNPH patients. Postoperatively, 90% of patients with presurgical bulbar dysfunction showed improvement, indicating that these symptoms are treatable and respond well to CSF shunt surgery.

The interpretation of corticobulbar symptoms in the elderly group requires caution, as dysphagia and dysarthria are not specific to iNPH. These symptoms can also arise from other etiologies, including pseudobulbar palsy following bilateral ischemic strokes (involving upper motor neurons, the posterior limb of the internal capsule, corona radiata, or brainstem) and various other age-related neurodegenerative diseases, particularly progressive supranuclear palsy. Nevertheless, the notable postoperative improvement of these bulbar symptoms following CSF shunt placement in our study strongly implicates iNPH as a primary, reversible etiology in these patients. This finding highlights the clinical necessity of incorporating iNPH into the differential diagnosis for elderly patients presenting with unexplained bulbar dysfunction, ensuring they are not prematurely excluded from potentially beneficial surgical interventions.

In a comparative study of depressive symptoms in iNPH patients, Israelsson et al. reported that symptoms of depression were considerably more common and more severe in iNPH patients compared with the control group [16]. Additionally, a case report described a patient whose clinical condition masqueraded as depression but was in fact iNPH; the patient’s depressive symptoms improved markedly following shunt surgery [8]. These findings indicate that psychiatric symptoms, although not typically recognized as a core clinical manifestation of iNPH, can be relieved after surgical intervention, supporting the importance of differentiating iNPH from primary psychiatric disorders.

As regards emotional dysfunction in iNPH, we hypothesize that ventricular enlargement disrupts frontal–subcortical circuits, particularly those involving the dorsolateral prefrontal cortex and basal ganglia. This disruption may result in symptoms such as emotional dysregulation (mood lability, apathy) and executive dysfunction (impaired concentration and psychomotor retardation). The improvement of these symptoms after shunt surgery supports the concept of functional impairment caused by altered CSF dynamics rather than irreversible structural damage.

With respect to quality of life, 82.9% of our patients regained independence in activities of daily living, and most were able to return to their premorbid routines. In addition to physical recovery, patients experienced psychosocial benefits, including enhanced self-confidence (observed in caregiver reports) and renewed optimism (patient self-assessments). These findings highlight that surgical success should not be judged solely by ventricular size reduction but also by improvements in functional independence, cognitive–emotional health, and psychosocial well-being. One patient in our cohort of 35 experienced CSF overdrainage resulting in chronic subdural hematoma. However, the prevalence of this complication in our study was considerably lower than the 9% rate reported in a previous study [11].

### Limitations

Several limitations should be acknowledged when interpreting the results of this study. First, regarding study design and patient population, this research was conducted as a single-center prospective cohort, which inherently limits external validity. Furthermore, the cohort exclusively comprises patients who presented with strong iNPH-like symptoms, demonstrated clinical improvement following a CSF tap test, and were ultimately selected for surgery. This creates a highly enriched, 'responder-like' subpopulation, meaning the findings may not be broadly generalized to all individuals with suspected iNPH and an Evans index below 0.3. Additionally, the lack of an age-matched control group restricts our ability to definitively differentiate disease-specific pathological changes from normal age-related variations.

Second, there were limitations related to our assessment methodologies. Radiologically, our study relied on the Evans index—a linear 2D measurement—rather than 3D volumetric analysis of the ventricles. While 3D volumetry may provide a more precise anatomical representation, it requires specialized software and dedicated neuroradiological expertise that are not universally available. From a practical standpoint, absolute ventricular volume does not strictly correlate with intraventricular pressure; thus, surgical decision-making remains primarily driven by clinical symptomatology combined with universally accessible indices like the Evans index. Clinically, our 6-month longitudinal design presented challenges such as participant fatigue, which resulted in missing data and inconsistent compliance, particularly for the video-recorded TUG assessments. Moreover, we utilized custom-developed assessment tools based on trichotomous outcome classifications (“improved,” “stable,” or “worse”). Although they were adapted from validated instruments with acceptable sensitivity, their ordinal, nonparametric nature relies heavily on patient or caregiver subjectivity, potentially introducing reporting bias. The variability in how different medical specialties interpret these tools could also limit their broader adoption.

Third, regarding follow-up and epidemiological data, the relatively short 6-month follow-up period precludes the assessment of long-term treatment durability and the identification of potential late-onset complications. Furthermore, formal epidemiological data regarding the exact prevalence of this specific patient subset remains lacking. Based on our institutional data in Fig. 2, 37 of 230 (16.1%) patients diagnosed with iNPH revealed an Evans index below 0.3. We strongly advocate that future prospective iNPH studies and registries systematically collect this demographic data to better define the epidemiological scope of these patients.

Despite these collective limitations, it is crucial to emphasize the clinical pragmatism underlying this study. We do not advocate for widespread screening or shunt surgery for all patients with normal ventricular size. Rather, our objective is to ensure that patients who clinically mirror the iNPH phenotype—including a positive physiological response to a tap test—are not denied potentially beneficial treatment simply because they do not meet classical radiological thresholds. These findings reinforce that iNPH diagnosis must remain primarily clinically driven, relying on comprehensive history taking and physical examination. When clinical and physiological assessments strongly align with the disease, the absence of ventriculomegaly or an Evans index below 0.3 should not absolutely preclude surgical intervention.

### Future directions

To address the limitations, future research should include multicenter studies with larger patient cohorts and randomized controlled trials to provide more robust evidence. Extended follow-up periods (≥ 12 months) are necessary to evaluate the long-term efficacy of treatment and detect delayed complications. The development and adoption of standardized protocols across institutions could enhance the reliability and generalizability of findings, while further validation of assessment tools would strengthen clinical utility.

## Conclusions

This study demonstrates that patients with clinically suspected iNPH and an Evans index below 0.3 can achieve satisfactory symptomatic improvement following CSF shunt surgery. These findings support the concept that early-stage iNPH may present before overt ventricular enlargement and that early intervention can yield a high response rate, as evidenced by both subjective reports and objective assessment scales.

Our results underscore the importance of broadening the diagnostic criteria for iNPH to include not only the classic triad but also balance disorders, dizziness, emotional symptoms, and bulbar dysfunction. In patients with an iNPH-like presentation, a positive spinal tap test, and supportive radiographic findings, even in the absence of an Evans index greater than 0.3, consideration for an iNPH diagnosis should be prompted. CSF shunt surgery should be strongly considered in such cases, as it may prevent disease progression and produce marked improvements in both symptoms and overall quality of life.

## Supplementary Information

Below is the link to the electronic supplementary material.

ESM 1 Supplementary Material 1 (DOCX 21.6 KB)

## Data Availability

No datasets were generated or analysed during the current study.

## Abbreviations

CSF: Cerebrospinal fluid
iNPH: Idiopathic normal pressure hydrocephalus
iNPHGS: Idiopathic normal pressure hydrocephalus grading scale
LPS: Lumboperitoneal shunting
TMSE: Thai Mental State Examination
TUG: Timed Up and Go
VPS: Ventriculoperitoneal shunting
