# Supplementary material for: Cerebrospinal fluid shunt outcomes in patients with idiopathic normal pressure hydrocephalus and an Evans index below 0.3: a prospective study
Source: Acta Neurochir (Wien). 2026 May 14;168(1):251. doi: 10.1007/s00701-026-06903-6 (PMC13719783; doi:10.1007/s00701-026-06903-6)
Supplement: Supplementary file 1 [file 701_2026_6903_MOESM1_ESM.docx]

**Supplementary Table 1** Idiopathic normal pressure hydrocephalus grading scale (iNPHGS) [11]

| **Grade** | | **Definition** |
| --- | --- | --- |
| Cognitive impairment | | |
|  | 0 | Normal |
|  | 1 | Complaints of amnesia or inattention but no objective memory and attentional impairment |
|  | 2 | Presence of amnesia or inattention but no disorientation to time and place |
|  | 3 | Presence of disorientation to time and place but conversation is possible |
|  | 4 | Disorientation to the situation or meaningful conversation impossible |
| Gait disturbance | | |
|  | 0 | Normal |
|  | 1 | Complaints of dizziness, drift, or dysbasia but no objective gait disturbance |
|  | 2 | Unstable but independent gait |
|  | 3 | Walking with any support |
|  | 4 | Walking not possible |
| Urinary disturbance | | |
|  | 0 | Normal |
|  | 1 | Pollakiuria or urinary urgency |
|  | 2 | Occasional urinary incontinence (1–3 or more times per week but less than once per day) |
|  | 3 | Continuous urinary incontinence (1 or more times per day) |
|  | 4 | Bladder function is almost or completely deficient |

Each domain is scored from 0 (normal) to 4 (most severe). Total iNPHGS score ranges from 0 to 12, with higher scores indicating greater overall symptom severity.
**Abbreviation:** iNPHGS, idiopathic normal pressure hydrocephalus grading scale

**Supplementary Table 2** Speech (vocalization) problem assessment, adapted from the Center for Neurologic Study–Bulbar Function Scale [12]

| **Grade** | **Definition** |
| --- | --- |
| 0 | Normal speech |
| 1 | Speech is slower than usual and hard to hear but the patient can complete sentences |
| 2 | Talking less because of the effort required to speak; repeated speech is sometimes required |
| 3 | Speech is difficult to understand; listeners who understand relay the patient’s words to others |
| 4 | Incommunicable; meaningless words or voice only |

Scored from 0 (normal) to 4 (most severe). Higher scores indicate greater vocalization impairment.

**Supplementary Table 3** Swallowing problem assessment, adapted from the Center for Neurologic Study–Bulbar Function Scale [13]

| **Grade** | **Definition** |
| --- | --- |
| 0 | Normal swallowing |
| 1 | Cutting food into smaller pieces makes it easier to chew and swallow |
| 2 | Gagging or choking after swallowing |
| 3 | Requires a soft diet at every meal to facilitate food intake |
| 4 | Feeding via nasogastric tube or previous history of aspiration pneumonia |

Scored from 0 (normal) to 4 (most severe). Higher scores indicate greater swallowing difficulty.

**Supplementary Table 4** Balance on standing problem assessment, adapted from the Hellström iNPH scale [14]

| **Grade** | **Definition** |
| --- | --- |
| 1 | Able to stand independently for more than 30 seconds on either lower extremity alone |
| 2 | Able to stand independently for less than 30 seconds on either lower extremity alone |
| 3 | Able to stand independently with feet together (at the heels) for more than 30 seconds |
| 4 | Able to stand independently with feet together for less than 30 seconds |
| 5 | Able to stand independently with feet apart (1 foot length) for more than 30 seconds |
| 6 | Able to stand independently with feet apart for less than 30 seconds |
| 7 | Unable to stand without assistance |

Scored from 1 (best balance) to 7 (most severe impairment). Lower scores indicate better standing balance. This scale is hierarchical; testing proceeds from grade 1 (most difficult) to grade 7 (least demanding).
**Abbreviation:** iNPH, idiopathic normal pressure hydrocephalus

**Supplementary Table 5** Emotion problem assessment, adapted from the Bipolar UK Mood Scale [15]

| **Grade** | **Definition** |
| --- | --- |
| 0 | Mood in balance; no symptoms of depression |
| 1 | Slight withdrawal from social situations; concentration less than usual; slight agitation |
| 2 | Feelings of panic and anxiety; concentration difficult and memory poor; some comfort in routine |
| 3 | Slow thinking; no appetite; need to be alone; sleep excessive or difficult; everything a struggle |
| 4 | Feelings of hopelessness and guilt; thoughts of suicide; little movement impossible to do anything |

Scored from 0 (no emotional symptoms) to 4 (most severe). Higher scores indicate greater emotional disturbance.

**Supplementary Table 6** Dizziness problem assessment, adapted from the AMER Dizziness Diagnostic Scale [16]

| **Grade** | **Definition** |
| --- | --- |
| 0 | No dizziness |
| 1 | Occasional dizziness (1–3 times monthly); related to body or head movement |
| 2 | Occasional dizziness (1–3 times weekly); related to body or head movement |
| 3 | Frequent dizziness not related to body or head movement; relieved by resting or sometimes requiring medication |
| 4 | Frequent dizziness not related to body or head movement; not relieved by resting or always requiring medication |

Scored from 0 (no dizziness) to 4 (most severe). Higher scores indicate greater dizziness severity. AMER refers to the developers of the scale.

**Supplementary Table 7** Barthel Index for activities of daily living [17–19]

| **Grade** | | **Definition** |
| --- | --- | --- |
| Bowels | | |
|  | 0 | Incontinent (or needs to be given enemas) |
|  | 1 | Occasional accident (once/week) |
|  | 2 | Continent |
| Bladder | | |
|  | 0 | Incontinent, or catheterized and unable to manage |
|  | 1 | Occasional accident (maximum once per 24 hours) |
|  | 2 | Continent (for over 7 days) |
| Grooming | | |
|  | 0 | Needs help with personal care |
|  | 1 | Independent with face, hair, teeth, and shaving (implements provided) |
|  | 2 |  |
| Toilet use | | |
|  | 0 | Dependent |
|  | 1 | Needs some help but can do something alone |
|  | 2 | Independent (on and off, dressing, wiping) |
| Feeding | | |
|  | 0 | Unable |
|  | 1 | Needs help cutting, spreading butter, etc. |
|  | 2 | Independent (food provided within reach) |
| Transfer | | |
|  | 0 | Unable; no sitting balance |
|  | 1 | Major help (1 or 2 people, physical); can sit |
|  | 2 | Minor help (verbal or physical) |
|  | 3 | Independent |
| Mobility | | |
|  | 0 | Immobile |
|  | 1 | Wheelchair independent, including corners |
|  | 2 | Walks with help of 1 person (verbal or physical) |
|  | 3 | Independent (but may use any aid, eg, walking stick) |
| Dressing | | |
|  | 0 | Dependent |
|  | 1 | Needs help but can do about half unaided |
|  | 2 | Independent (including buttons, zips, laces, etc) |
| Stairs | | |
|  | 0 | Unable |
|  | 1 | Needs help (verbal, physical, or carrying aid) |
|  | 2 | Independent up and down |
| Bathing | | |
|  | 0 | Dependent |
|  | 1 | Independent (or in shower) |

Total score ranges from 0 to 20, with higher scores indicating greater independence in activities of daily living. Most domains are scored from 0 to 2; transfer and mobility are scored from 0 to 3; grooming and bathing are scored from 0 to 1.
